# Techniques for Screening Translation Inhibitors

**DOI:** 10.3390/antibiotics5030022

**Published:** 2016-06-24

**Authors:** Ilya A. Osterman, Alexey A. Bogdanov, Olga A. Dontsova, Petr V. Sergiev

**Affiliations:** Department of Chemistry and A.N. Belozersky Institute of Physico-Chemical Biology, Lomonosov Moscow State University, Moscow 119992, Russia; osterman@yandex.ru (I.A.O.); bogdanov@genebee.msu.ru (A.A.B.); olga.a.dontsova@gmail.com (O.A.D.)

**Keywords:** translation, ribosome, antibiotic, high throughput screening

## Abstract

The machinery of translation is one of the most common targets of antibiotics. The development and screening of new antibiotics usually proceeds by testing antimicrobial activity followed by laborious studies of the mechanism of action. High-throughput methods for new antibiotic screening based on antimicrobial activity have become routine; however, identification of molecular targets is usually a challenge. Therefore, it is highly beneficial to combine primary screening with the identification of the mechanism of action. In this review, we describe a collection of methods for screening translation inhibitors, with a special emphasis on methods which can be performed in a high-throughput manner.

## 1. Introduction

Each year the number of resistant bacterial strains increases, so finding new antibiotics is one of the principal goals of modern biochemistry. Both natural extracts and chemically synthesized compounds can be used in the high-throughput screening of antimicrobial agents [1]. These methods of screening are widely used and usually new inhibitors of growth are found, but understanding the mechanism of action is a more sophisticated task. The majority of known antibiotics affect translation. Conservation of the bacterial ribosome structure, which is at the same time significantly different from a eukaryotic ribosome, makes the majority of these antibiotics suitable for clinical use [2]. There are many methods for studying translation inhibitors: some of them can just indicate the fact of protein synthesis inhibition, while others can provide information about particular changes in ribosome activity. In this review, we summarize the methods of screening for translation inhibitors and of confirming their mechanism of action. Some of these methods could be used at a very early stage of finding new antibiotics, namely at the stage of high-throughput screening, and other methods help to test just several potential candidates while giving much more information about the mode of action.

## 2. Biosensor Assays

The most effective known methods for initial screening are to use biosensor bacterial strains with different reporter systems detecting the presence of specific antibiotics. (Table 1). While some of these methods aim at fishing out a wide range of compounds that act on translation, other methods are useful in identifying the presence of a specific, narrow group of translation inhibitors.

### 2.1. Real-Time Measurement of Protein Inhibition Using a Luciferase Assay 

Three different luciferase systems—*lucGR* from *Pyrophorus plagiophthalamus*, *luxAB* from *Photorhabdus luminescens*, and *luxAB* from *Vibrio harveyi*—were cloned under the main leftward promoter pL from bacteriophage λ [3]. This promoter was induced after a temperature upshift to 42 °C. The induction was made after or before adding the test substances, and the difference in luciferase activity was measured. This allowed the authors to estimate the direct effect of tested chemicals on protein synthesis in real time. The luciferase gene *Pyrophorus plagiophthalamus* was found to be the best measure among these three test systems. The presence of translation inhibitors, such as tetracycline and chloramphenicol, was vividly shown by this method. The authors also showed that with the use of lyophilized bacteria, the test could be done in one hour without cultivation. The replacement of a strong pL promoter by an inducible tetracycline promoter allowed the authors to significantly increase the sensitivity of transcriptional and translational antibiotic detection [4]. These assays could be carried out in a high-throughput manner. The disadvantage of this method is that it is an “Off” assay, with the loss of the detection signal measured. Compounds interfering with energy generation or non-translation antibiotics, which affect protein synthesis in an indirect way (for example, rifampicin and general toxicants such as mercuric chloride and phenol), will also be detected by this approach, and it is impossible to find the difference between translation inhibitors and antibiotics affecting protein synthesis indirectly. 

### 2.2. Stress Response Assay

An antibiotic’s action on a cell typically induces a specific stress response, which can be used to classify an antibiotic’s mode of action [5]. Temperature upshifts, or exposure to low temperatures, dramatically change the range of synthesized proteins, and the action of ribosomal antibiotics also could lead to the expression of cold (C-antibiotics chloramphenicol and tetracycline) or heat (H-antibiotics streptomycin and neomycin) shock proteins, even when *E. coli* is cultivated at 37 °C [14]. To create ribosomal antibiotic sensors, the 5’UTR promoter and the beginning of the coding region of *ibpA* and *cspA* genes were placed upstream to the *lacZ* gene and these constructs were integrated into the genome of wild-type *E. coli* K-12. CspA is major cold shock protein of *E. coli* [15], and due to the difference in the secondary structure of the 5’ part of mRNA under normal and low temperatures, the expression rate of *cspA* increases dramatically in the conditions of cold shock. The expression of *cspA::lacZ* fusion was activated as expected by chloramphenicol and tetracycline. IbpA is a small heat shock protein and its expression increases significantly under temperature upshift; the expression of *ibpA::lacZ* fusion is induced by streptomycin, neomycin (aminoglycoside antibiotics) and polymyxin B (non-ribosomal antibiotic). Both reporters can be used in a liquid culture and on agar plates.

The inactivation of the *tolC gene* [6] in these reporter strains decreases the concentration of the antibiotic, which could be detected. This is important for the adaptation of this assay to an actual search for new antibiotics. The further replacement of *lacZ* with *luc* (*Photinus pyralis* luciferase) [7] makes the reporter faster and more sensitive, and gave the authors the ability to detect two different antibiotics, with the use of both *lacZ* and *luc* in one biosensor strain.

### 2.3. Tetracycline Detection Assay

One specific detection system is based on the tetracycline response element from transposon Tn10 plus two different bioluminescence systems (the bacterial luciferase operon of *Photorhabdus luminescens* and firefly *Photinus pyralis* luciferase) [8]. A tetracycline-sensitive promoter and a tetracycline repressor gene were cloned upstream of *luc* genes on a plasmid. In the absence of tetracycline, the repressor protein binds the promotor and inhibits expression. The binding of tetracycline to the repressor leads to dissociation of its complex with DNA, and activates downstream gene expression. Both sensors can be used for the detection of tetracyclines in veterinary samples, or for the screening natural compounds. The quality of sensors is not shown to decrease through lyophilization and freezing. These sensors are very sensitive (10 ng), but can detect only one class of antibiotics.

### 2.4. Macrolide Detection: mphA Inducible Assay 

MphA is a macrolide 2′-phosphotransferase. This enzyme inactivates 14-membered macrolides, such as erythromycin. The expression of *mphA* is dependent on the presence of macrolides. MphR is a transcriptional repressor of *mphA*. The binding of a macrolide to MphR leads to a dissociation of the repressor from the *mphA* promoter, and expression increases. An *mphA* promoter was placed upstream to the luciferase genes of *Vibrio fischeri,* and made the biosensor dependent on the presence of classical 12- and 14-membered macrolides, or semisynthetic clarithromycin and azithromycin [9]. This assay helps to differentiate macrolides from other antibiotics, and even from other translation inhibitors. It can be performed in liquid media or on agar plates, and can be used for high-throughput screening for macrolides.

### 2.5. Macrolide Detection: ermC Inducible Assay 

*ermC* is a specific RNA methyltransferase which is expressed in response to macrolide exposure [16] and modifies the A2058 nucleotide. The leader peptide *ermC(L)* was placed upstream of the *lacZ* gene, which made the expression of the reporter gene dependent on the presence of macrolides and ketolides [10]. By means of this reporter, it is possible to detect cladinose containing 14-member-ring macrolides and ketolides, but not 16-member-ring macrolides, chloramphenicol or azithromycin. This approach can be adapted to high-throughput screening. Together with the previous assay, these methods can help not only to detect macrolides, but also to differentiate between them.

### 2.6. Attenuation-Based Dual Fluorescent Reporter Assay 

A broad-specificity system for the detection of translation inhibitors was developed on the basis of the tryptophan attenuator *trpL* and cerulean fluorescent protein [11]. Two tryptophan codons were replaced by two alanine codons, which made the expression of the signal gene independent of the tryptophan concentration. At normal conditions, fast translation of the modified leader peptide leads to transcription termination and inefficient expression of the downstream reporter gene. However, the stalling of the ribosome due to translation inhibition relieves the attenuation, and leads to the activation of cerulean fluorescent protein gene expression. By means of these sensors, it is possible to detect ribosomal antibiotics such as erythromycin, tylosin, azithromycin, chloramphenicol, linezolid and lincomycin. The possibility of using the *tolC* knock-out strain with this reporter system was demonstrated. The minimal detectable concentration of the tested substances decreased significantly. A new translation inhibitor, amicoumacin A, was identified by means of this reporter system [17]. This approach can be used in a high-throughput manner on agar plates with a reporter strain. 

### 2.7. Panel of Reporter Strains that Lack Antibiotic Resistance 

The majority of antibiotic sensor strains have one significant drawback: they are resistant to at least one antibiotic used to maintain a reporter plasmid. This method is not useful for broad screening, since some potential antibiotics will be missed. A tryptophan auxotroph *E. coli* strain helps to avoid the antibiotic resistance marker. Further, 14 promoters (*emrA, acrA, zwf, soxS, tolC, inaA, zntA, marR, recA, micF, katG, sodA, rpoB and ompF*) were placed upstream to the *luxCDABE* operon, and 14 reporter strains were created [12]. The authors tested the action of 11 antibiotics on these 14 reporter strains. As a result, different mechanisms of action could be discriminated. The *soxS* reporter was specifically induced in the presence of the ribosomal antibiotics tetracycline, oxytetracycline and chloramphenicol. The exact mechanism linking this reporter with translation inhibition is still unknown, but empirically it can be used for the detection of at least two types of translation inhibitors.

### 2.8. Biosensor for Translational Antibiotics Based on Bacillus subtilis yheI Gene 

Promoters of several *B. subtilis* genes were cloned upstream to the firefly luciferase gene, and these reporters were tested with a large panel of the antibiotics with various mechanisms of action. The *yheI*-based reporter [13] specifically reacts with protein-biosynthesis inhibitors such as linezolid, doxycycline, fusidic acid and chloramphenicol. The molecular mechanism linking translation with *yheI* gene expression is still lacking. This protein has a similarity to multidrug-resistant ABC pump proteins. In spite of the absence of a complete understanding of the mechanism, this reporter can be used in screening new potential antibiotics.

### 2.9. Transcriptional Sensors Based on Promoters Library 

The DNA of *Salmonella typhimurium* was fragmented and cloned upstream of *luxCDABE* in order to produce a library of more than 6000 individual “promoter clones” [18]. For a large part of the individual clones, the specific induction or inhibition by translational (erythromycin) and transcriptional (rifampicin) inhibitors at a low concentration was detected. When a strain resistant to a particular antibiotic was used as a host for the set of reporter plasmids, the pattern of ”promoter clones” responding to an antibiotic changed dramatically. For several promoters, the specificity of the induction of expression to different antibiotics was shown. This method allows the detection of known natural compounds at low concentrations in a high-throughput manner. The described approach could be used for producing biosensors for various antibiotic classes.

## 3. In Vitro Methods

After finding a new translation inhibitor by means of any reporter assay, an in vitro confirmation of the mechanism of action is necessary. There is a broad spectrum of various in vitro techniques. 

### 3.1. In vitro Protein Synthesis Inhibition Assay

Inhibition of in vitro translation is the easiest way to indicate the mechanism of action of a potential translation inhibitor. Such a reaction can be carried out in a coupled transcription/translation reaction, either supplied by DNA or in a translation reaction supplied by mRNA. Either cell extracts [19] or pure components [20] can be used as a source of translation components. The products of such translation can be visualized by incorporation of radioactive or fluorescent amino acids. The signal reduction in the presence of the substances tested verifies that the antibiotic affects protein synthesis.

To simplify the procedure of measurement, to avoid the purification of the synthesized protein or the separation of proteins in gel, luciferase mRNA was used. Adding the luciferin after a reaction allows the detection of luminescence, and measures translation inhibition. 

The significant reduction of the reaction volume to 2 μL allowed the authors to use this in vitro inhibition assay for high-throughput screening [21]. By means of this assay, 30,000 compounds were tested on 1536-well microplates. As a result, several new translation inhibitors were found. 

### 3.2. Toe-Printing of Antibiotic-Stalled Ribosomes 

The mRNA-ribosome complex can be analyzed by means of reverse transcription with an oligonucleotide annealed downstream to a bound ribosome; the stopping of reverse transcriptase indicates the location of the ribosome on the mRNA. Various translation antibiotics inhibit specific functions of ribosomes, and the site of an arrested ribosome is dependent on the mechanism of the antibiotic’s action. A toe-printing assay can be done directly in a translation reaction in a cell-free transcriptional/translational system composed of purified components. A large set of translation inhibitors affecting the 50S and 30S subunits were tested, and specific patterns of reverse transcription stops were detected [22]. Some antibiotics, such as thiostrepton, clindamycin, tiamulin and pristinamycin IIB, arrest a ribosome on a start codon and could not be distinguished by this method. All of these antibiotics were acting on a large subunit. Another antibiotic group acting on a small subunit, such as streptomycin, kasugamycin and edeine, could not be distinguished because it suppresses all stops of reverse transcriptase. Fortunately, chloramphenicol, florfenicol, linezolid, erythromycin, telithromycin, tylosin, evernimicin, pristinamycin IB, spectinomycin, kanamycin, tetracycline, pactamycin and gentamicin produce a unique, distinguishable pattern of arrested ribosomes. It was shown that this approach is useful for discrimination between known antibiotics while screening culture broths of natural antibiotic producers. Application of this system allows a researcher to avoid the purification and chemical identification of known antibiotics from newly identified producer strains. 

### 3.3. SPARK-Sensitive Method for Monitoring Peptidyl Transferase Activity 

Peptide bond formation is a key stage in protein synthesis. If one participant of peptidyl transferase reaction is tritiated and the other is biotin-tagged, the efficiency of peptide bond formation can be easily measured [23]. The decrease of a signal in the presence of a tested antibiotic confirms that it is a translation inhibitor. The SPARK method can be carried out in various versions: tritiated formyl-Met-tRNA at the P-site, and biotin-puromycin or Lys-tRNA biotinylated at the side-chain amino group of lysine, or unlabeled N-biotinylated Phe-tRNA and tritiated Phe-tRNA as P- and A-site substrates for poly(U)-programmed 70S ribosomes. This system also helps to distinguish different ribosomal antibiotics between themselves: chloramphenicol, sparsomycin, hygromycin A, carbomycin and lincomycin (peptidyl transferase inhibitors) inhibited formation of the reaction product, while at the same time, erythromycin, a macrolide which does not inhibit transpeptidation, did not decrease the amount of the reaction product. The simplicity of this method, due to streptavidin-coated polymeric scintillation proximity assay beads containing an embedded scintillant, provides the possibility to use it directly in screening new peptidyl transfer inhibitors.

### 3.4. Antibiotic Binding to a Fluorescently Labeled Ribosome 

The insertion of a fluorescent label into a ribosome provides the possibility to study the binding of small molecules such as antibiotics. Such a fluorescent label was added to the S12 protein near the drug’s binding site. The binding of the antibiotic changed the environment of the fluorophore and changed the fluorescence [24]. For streptomycin and neomycin, the constant of dissociation could be measured by this method, and the results are in agreement with those previously detected by other methods. This method could be used for the confirmation and study of the binding of antibiotics at known places on ribosome.

### 3.5. The Influence of an Antibiotic on Ribosomal Assembly Measured by Means of Fluorescenly Labeled Ribosomal Subunits

Both large and small ribosomal subunits were labeled with fluorescent proteins EGFP and mCherry, respectively [25]. Microscopic analyses and direct measurements of the fluorescence of sucrose gradient fractions provided the possibility to estimate the subunit assembly rate. The absence of proteins involved in ribosomal assembly led to dramatic changes in fluorescent signals on the gradient, while at the same time the influence of translation inhibitors erythromycin and chloramphenicol on the fluorescence signal of the ribosome was very small, in spite of their strong effect on growth. These antibiotics inhibit translation but do not affect ribosomal assembly. This system could be used to detect ribosome assembly inhibitors.

### 3.6. The Kinetics of Translocation as Sensor of Antibiotic Mechanism of Action

Several antibiotics which affect translocation, such as viomycin, spectinomycin, streptomycin and hygromycin B, could be detected by observation of translocation kinetics [26]. Labeling MetPhe-tRNA, located in the P-site and mRNA, makes it possible to independently monitor the influence of antibiotics on the translocation at 30S and 50S subunits. This approach provides the possibility to distinguish between various translation antibiotics according to their effect on translocation. These differences are in agreement with the actual mechanism of an antibiotic’s mode of action. This method could not be used for primary screening, but it might be useful for further analysis of newly discovered ribosomal antibiotics.

### 3.7. Biotinylated Biosensors Based on ELISA Technology

There are proteins which can specifically bind certain antibiotics, and usually these proteins regulate the expression of resistance genes. Based on these proteins, specific biosensors were produced [27]. Biotinylated DNA operator sequences, bound to streptavidin plates, were incubated with His_6_-tagged biosensor proteins. Following the addition of tested substances, the case of a specific interaction of an antibiotic with the protein leads to dissociation of the protein complex with immobilized DNA. The amount of the biosensor protein bound to the immobilized DNA was detected by antibodies in a specific ELISA assay. This sensor system was created to detect tetracycline, streptogramin and macrolide antibiotics in different solutions, including milk and serum at ng/mL concentrations. This approach is very sensitive, but allows the detection of only one antibiotic, and could not be used in *de novo* screening.

## 4. In Vivo Methods

The inhibition of protein synthesis leads to strong changes in the majority of cell processes, but for different antibiotics the effect is different. Studying changes in synthesized proteins, cell shape and the ratio between cell metabolites can help us to classify an antibiotic’s mode of action.

### 4.1. Fluorescent Microscopy and Bacterial Cytological Profiling 

By means of specifically labeling bacterial DNA and the cell membrane, it is possible to characterize the bacterial state by the shapes of the membrane and the nucleoid form. If performed after adding an antibiotic, specific changes will be detected [28]. This method allows the indication of the cellular pathway affected by an antibiotic. Various antibiotics were tested with this method, and specific cytological profiles were obtained. Translation antibiotics showed a different profile, so this method could be used for the detection of new translation inhibitors. Interestingly, this approach allowed the authors to distinguish between different ribosomal inhibitors. For example, peptide chain elongation inhibitors such as tetracycline and chloramphenicol demonstrated a different cytological profile than aminoglycosides, and puromycin, which leads to premature protein chain termination, causes yet more characteristic cytological changes. 

### 4.2. Proteomic Signature 

The addition of an antibiotic leads to specific changes in proteomes, so the study of proteomic changes could also be used for the identification of the mechanism of action of new antibiotics [29]. Two-dimensional (2D) protein gel is one of the standard tools for studying such changes. The effects of 40 different antibiotics on the *B. subtilis* proteome were measured [30]. There are “marker proteins” for different antibiotics, the concentrations of which increased after treatment. Different translation inhibitors even showed different patterns of altered proteins. Inhibitors of peptidyl-transferase induce the expression of proteins involved in translation, while at the same time, the inhibition of A-site binding by chloramphenicol or the blocking of a ribosomal tunnel by erythromycin induces different proteomic signatures. Aminoglycosides cause errors in translation, and their addition led to an increased expression of chaperons and proteases. The inhibitors targeting aminoacyl-tRNA synthetases increased the proteins involved in amino acid biosynthesis. Interestingly, puromycin again fell into an individual group—initially increasing the concentration of chaperons and proteases, but after 40 min of treatment only small proteins could be fully translated. 

The 2D protein gel can be used for analysis of the *de novo* proteome by means of a radio-labeled or fluorescent-labeled amino acid [31], while using fluorescence allows one to carry out 2D difference gel electrophoresis and compare, in one gel, the *de novo* proteome of the cells treated with an antibiotic with that of untreated cells [32].

### 4.3. The Mechanism of Resistance as a Sensor of the Mechanism of Action

Antibiotic resistance is usually a significant problem in antibacterial treatment, but it also could be used for the identification of the mechanism of action of a new antibiotic. The cultivation of bacteria at the inhibitory concentration of a tested antibiotic leads to the appearance of resistant mutations, and the following sequencing of their genomes gives us information about the mutant genes, and can help researchers understand the mechanism of action. For searching translation inhibitors, a mutant strain with only one ribosomal operon was created, and the chance of the appearance of a resistant mutation in ribosomal RNA in such a strain is much larger [22]. The location of the mutation usually indicates the place of antibiotic binding, and further footprinting analysis should prove it. This approach can be used for determining the mechanism of action of new antibiotics or for the screening of natural substances for rejecting known antibiotics. 

### 4.4. Phenotypic Profiling by Raman Spectroscopy

Raman spectroscopy can be used for phenotypic profiling of bacterial cells treated with a different class of antibiotics [33]. Cells treated with protein synthesis inhibitors demonstrating specific Raman spectra differed from that of cell wall, DNA and RNA synthesis inhibitors. Based on data from various antibiotics with different mechanisms of action, a predictor algorithm was created, which could be effectively used to determine the class of the antibiotic.

## 5. Conclusions

**Figure 1:**
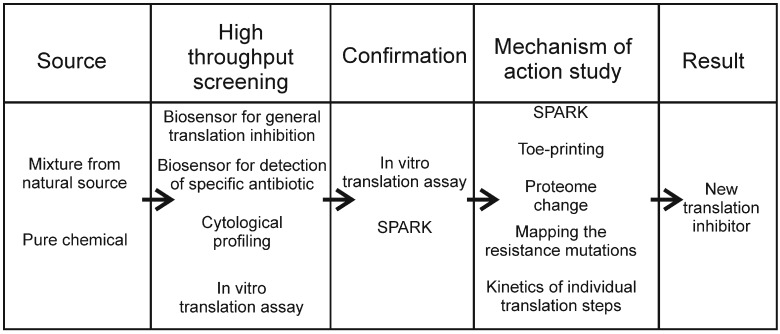
The principal scheme of translation inhibitor screening.

There are many good methods for studying translation inhibitors, and any of them can be used for studying a particular compound. However, only a subset of methods can efficiently be used for screening thousands of compounds with the aim of finding a new antibiotic targeting translation. Based on this study, one principal scheme of translation inhibitor screening could be suggested (Figure 1). Reporter assays are the best candidates for initial high-throughput screening, due to the ease of automation and rapidity. Since there is no need for precise information about minimal inhibitory concentrations (MIC) of tested substances during a first screening, the assays should be carried out on agar plates rather than in liquid media, since unlike in liquid, where it is necessary to test several concentrations to determine the MIC, spotting of tested compounds on agar plates creates a gradient between lethal and sublethal concentrations. Reporter assays are very useful for screening natural extracts, because such assays do not generally require an active compound to be purified. Following the initial identification of a mixture containing a translation inhibitor using a reporter assay, it is also easy to fractionate the mixture and test fractions after separation of an extract. When in vitro confirmation of translation inhibition is necessary, it can be done by translation in cell extract or with pure components. The next step is to reveal the particular influence of antibiotics on translation. Mapping of antibiotic resistance mutations in rRNA genes, or in other parts of the genome, can help to identify an antibiotic binding site or indicate the stage of translation which is affected by the antibiotic. Toe-printing and kinetic studies of individual translation steps could add the information about the mechanism of action. The proteomic studies could help us to understand the mechanism of action and show the effect of antibiotics on intracellular process. Other methods described in this review could also be used for a more detailed characterization of an antibiotic’s mode of action. 

## Figures and Tables

**Table 1:** Biosensors for translation inhibitors. If an antibiotic is correctly detected by a biosensor, it is marked as P (positive); if it is not detected and should not be detected—N (negative); if it is detected but should not be detected—FP (false positive); if it is not detected, but should be detected—FN (false negative).

Type	Year, Reference	Signal	Assay Time and Conditions	Description	Tested Molecules	Notes
Biosensor for translation inhibitors	1995, [3]	Luciferase	30–45 min (liquid assay)	Plasmid reporter, *E. coli* pL (bacteriophage lambda leftward promoter)–*luxAB (Photorhabdus luminescens)* pL–*luxAB* (*Vibrio harveyi*) pL–*lucGR* (*Pyrophorus plagiophthalamus)*	Tetracycline P, Sodium fluoride FP, Chloramphenicol P, Nicotine FP, Cycloheximide N, Mercuric chloride FP, Polymixin B FP, Phenol FP	Direct measuring of translation
Biosensor for translation inhibitors	2003, [4]	Luciferase	Up to 3 h (liquid assay)	Genome reporter, *E. coli* p*tetA* (tetracycline inducible)–*luxCDABE (P. luminescens)*	Rifampicin FP, Imipenem FP, Chloramphenicol P, Sulfadiazine FP, Ciprofloxacin FP, Cefotaxime FP, Erythromycin P, Spiramycin P, Trimethoprim FP	Direct measuring of translation
Biosensor for translation inhibitors	1999, [5]	Beta-galactosidase	Up to 3 h (liquid assay) Overnight (solid assay)	Genome reporter, *E. coli or E. coli:ΔtolC* [6] , p*cspA* (promoter-utr-start of codon region of *cspA* gene)–*lacZ or luc (Photinus pyralis)* [7] p*ibp* (promoter-utr-start of codon region of *ibp* gene)–*lacZ* *or luc (Photinus pyralis)* [7]	Tetracycline P, Chloramphenicol P, Streptomycin P, Neomicin P, Polymyxin B FP, Nalidixic acid N, Carbenicillin N	Stress response promoters
Biosensor for tetracyclines	2000, [8]	Luciferase	90 min (liquid assay)	Genome reporters, *E. coli*, p*tetA*-luc (*Photinus pyralis*) p*tetA*-*luxCDABE* (*Photorhabdus luminescens*)	Tetracyclines P	Tetracycline inducible promoter of *tetA*
Biosensor for macrolides	2007, [9]	Luciferase	2 h (liquid assay) 1–2 h (solid assay)	Genome reporter, *E. coli* p*mphR (A)* (*mphA* promoter)–*luxCDABE* (*Vibrio fischeri*)	Erythromycin P, Methymycin P, Azithromycin P, Clarithromycin P, Oleandomycin P, Tetracycline N, Chloramphenicol N, Gentamycin N, Cefaclor N, Novobiocin N, Fosfomycin N, Vancomycin **N**	Macrolide inducible promoter of *mphA*
Biosensor for macrolides	2008, [10]	Beta-galactosidase	Overnight (solid assay)	Plasmid reporter, *E. coli*, P*tac*-*ermC (L)*–*lacZ*(α).	Erythromycin P, Clarithromycin P, Azithromycin FN, Clarithromycin P, Josamycin N, Telithromycin P, Spiramycin N, Tylosin N, Roxithromycin P, Chloramphenicol N	Macrolide inducible expression of *ermC*
Biosensor for ribosome stalling inhibitors	2012, [11]	Cerulean fluorescent protein	Overnight (solid assay) Overnight (liquid assay)	Plasmid reporter, *E. coli or E. coli:ΔtolC*, pT5–*trpL*–2Ala–CER.	Erythromycin P, Clarithromycin P, Roxithromycin P, Oleandomycin P, Tylosin P, Azithromycin P, Chloramphenicol P, Linezolid P, Lincomycin P, Sulfanilamide N, Polymyxin N, Rifampicin N, Kanamycin N, Tetracycline FN, Streptomycin N, Vancomycin N, Clindamycin FN, Nalidixic acid N, Phosphomycin N, Levofloxacin N, Norfloxacin N, Tobramycin N, Neomycin N	Ribosome stalling induces expression of the signal gene
Biosensor for translation inhibitors	2012, [12]	Luciferase	10 h (liquid assay)	Plasmid reporter, *E. coli* p*soxS* (promoter of *soxS* gene)–*luxCDABE (Photorhabdus luminescens)*	Tetracycline P, Oxytetracycline P, Chloramphenicol P, Sulfamethoxazole N, Sulfadimethoxine N, Ampicillin N, Amoxicillin N, Nalidixic Acid N, Rifampicin N, Puromycin FN, Colistin N	
Biosensor for translation inhibitors	2007, [13]	Luciferase	4 h (liquid assay)	Plasmid reporter, *Bacillus subtilis* p*yheI* (promoter of *yheI* gene)–luc (*Photinus pyralis*)	Moxifloxacin N, Ciprofloxacin N, Trovafloxacin N, Nalidixic acid N, Novobiocin N, Trimethoprim N, Azaserine N, Mitomycin C N, Streptovaricin N, Rifampicin N, Linezolid P, Doxycycline P, Fusidic acid P, Chloramphenicol P, Gentamicin FN, Kanamycin FN, Puromycin FN, Actinonin FN, Cefoxitin, Oxacillin N, Mersacidin N, Ramoplanin N, Vancomycin N, Tunicamycin N, Polymyxin B N, Moiramide B N, Cerulenin N, Triclosan N, Netropsin N, Ethidium bromide N, Actinomycin D N, Monensin N, N-ethyl maleimide N	
